# Association between polycyclic aromatic hydrocarbons exposure with red cell width distribution and ischemic heart disease: insights from a population-based study

**DOI:** 10.1038/s41598-023-50794-x

**Published:** 2024-01-02

**Authors:** Pin Wu

**Affiliations:** Department of Hematology, Jiangnan University Medical Center, No. 68 Zhongshan Road, Wuxi, Jiangsu China

**Keywords:** Environmental sciences, Risk factors

## Abstract

This study investigates the association between polycyclic aromatic hydrocarbon (PAH) exposure, red blood cell distribution width (RDW), and ischemic heart disease (IHD) in a sample of 3003 participants from the National Health and Nutrition Examination Survey (NHANES). We hypothesize that RDW may mediate the effect of hydroxylated PAHs (OH-PAH) on IHD. Logistic regression models reveal significant associations between increased urinary PAH metabolite concentrations and IHD, as well as positive associations between PAH metabolites and RDW. Weighted Quantile Sum (WQS) regression and Bayesian Kernel Machine Regression (BKMR) analyses confirm the significant associations of the OH-PAH mixture with IHD and RDW. Mediation analysis demonstrates that RDW partially mediates the relationship between PAH exposure and IHD, accounting for 2–4.6% of the total effects. Our findings highlight the potential underlying mechanisms linking PAH exposure, RDW, and IHD and emphasize the importance of addressing environmental pollutants like PAHs in maintaining cardiovascular health and informing public health policies.

## Introduction

Polycyclic Aromatic Hydrocarbons (PAHs) are a group of organic compounds consisting of multiple aromatic rings characterized by their stable, flat, and rigid molecular structure^1^. The ubiquity of PAHs is primarily driven by human activities, leading to a profusion of sources that release these compounds into the atmosphere. The United States Environmental Protection Agency (USEPA) has identified over 100 PAHs, designating 16 as priority contaminants due to their toxic nature^2^. Major sources of PAH emissions include vehicle exhaust, industrial processes, residential heating, and natural sources like wildfires and volcanic eruptions^1^. Humans can be exposed to PAHs through various pathways, including inhalation, skin contact, and ingestion^3–5^. The impact of PAHs on human health depends on various factors such as exposure duration, route, concentration, and PAH toxicity^6^. Many PAHs are considered carcinogenic, mutagenic, and teratogenic, posing significant risks to human health, and the primary health concern from PAH inhalation exposure is an increased likelihood of lung cancer^6^.

Environmental pollutants have profound effects on human health, with Ischemic Heart Disease (IHD) being a principal concern. IHD, which is the leading cause of death globally and a major cause of disability worldwide^7,8^, results from reduced blood supply to the heart muscle due to narrowed or blocked coronary arteries^9^. The relationship between air pollution and heart disease (HD) has been reported by many studies ^10–12^. Intriguingly, emerging evidence pinpoints polycyclic aromatic hydrocarbons (PAHs) as potential risk factor in the onset of IHD. Associations have been drawn between PAH exposure and heart rate variability (HRV)^13,14^, with the latter being recognized as a central pathophysiologic mechanism behind detrimental cardiac events^15^. Notably, the presence of PAH metabolites in urine correlates with an elevated risk of cardiovascular ailments^16^. In occupational settings, PAH exposure has been ominously linked to fatal IHD outcomes, showcasing a discernible exposure–response relationship^17^. Furthermore, evidence suggests that particulate matter (specifically PM3.5) laden with PAHs can exacerbate the risk of IHD^18^, emphasizing the pervasive threat of these compounds in various facets of daily life. While the exact mechanistic pathway remains to be fully delineated, PAHs have been recognized for their potential to initiate and promote oxidative stress, which can cascade into cardiovascular pathologies (Li et al., 2008). This oxidative stress is believed to initiate inflammatory processes, culminating in endothelial dysfunction—a key contributor to the development of atherosclerosis and, subsequently, IHD (Poznyak et al., 2020).

In recent research developments, Red Cell Width Distribution (RDW) has been identified as a significant biomarker in predicting cardiovascular diseases^19–23^. Elevated RDW levels, which indicate increased variability in red blood cell distribution width, have been associated with a higher risk of adverse outcomes in various cardiovascular conditions, including heart failure, myocardial infarction, and peripheral artery disease^24^. The mechanisms linking RDW and cardiovascular diseases are not yet fully understood, but factors such as inflammation, oxidative stress, and impaired iron metabolism may play a role^21^.

While numerous studies have highlighted the link between PAH exposure and IHD incidence, and some have identified RDW as a biomarker for heart-related diseases, to the best of our knowledge, no study has delved into the interrelationship between RDW, PAH exposure, and IHD. Unraveling this tripartite relationship is of significant importance, and a deeper understanding of these interactions could reveal insights of how PAH cause cardiovascular disease and may pay the way for therapeutic strategies against pollutants induced IHD. Furthermore, considering the ubiquity of PAHs in our environment and the widespread prevalence of IHD, understanding any mediating role RDW might play could have profound implications for public health strategies and policymaking, emphasizing the importance of monitoring RDW levels and regulating environmental exposures, like PAH, to enhance cardiovascular health.

**Figure 1:**
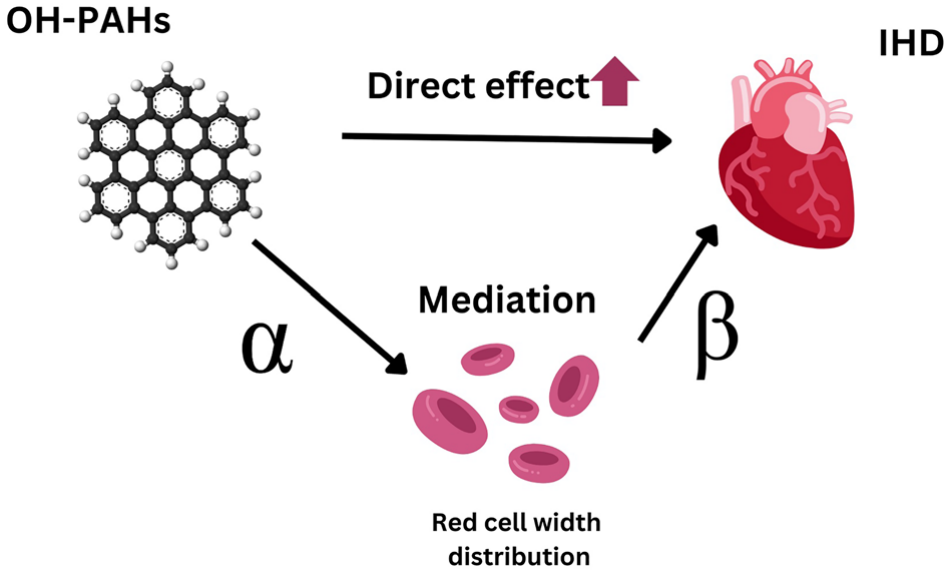
Hypothesized relationship among PAH, RDW, and IHD.

In this study, we seek to elucidate the relationship between PAH exposure, RDW, and IHD, contributing novel insights into potential underlying mechanisms. Our hypothesis suggests that RDW may mediate the effect of hydroxylated PAHs (OH-PAH) on IHD (Fig. 1). Investigating this potential pathway could shed light on novel risk factors and preventive measures for IHD, highlighting the importance of addressing environmental pollutants like PAHs in maintaining cardiovascular health. This study seeks to fill the existing knowledge gap and may contribute to the development of informed public health policies aimed at mitigating the impact of PAH exposure on IHD risk.

## Materials and methods

### Study subjects

The National Health and Nutrition Examination Survey (NHANES) employed a sophisticated probability sampling approach to ensure national representation of the non-institutionalized civilian population in the United States. Data for this study were extracted from the NHANES conducted between 2013 and 2016. Each NHANES cycle's subsample accurately represented the overall population. The appropriate weight was calculated considering the additional sampling step, unequal selection probability, and non-response rate^25^.

As depicted in Figure S1, 18,587 participants were enrolled across four survey cycles from 2013 to 2016. Exclusions were made for individuals under 20 years (n = 7525) and those with missing data in confounding variables (n = 1,311), resulting in 9751 respondents. Among these, 6,619 had incomplete data for one or more OH-PAH variables, and 129 had missing values for RDW and IHD. Ultimately, an analytic sample of 3,003 participants with complete data of interest was included in this study. Despite the subset of 3003 participants representing only 16% of the entire sample, the distributions of demographic variables like age, sex, poverty-income ratio, education, and race did not show significant differences. To test the distribution differences between subsample and original dataset, Kolmogorov–Smirnov tests were used for continuous variables and chi-square tests were used for categorical variables (Table S1).

### Urinary OH-PAHs assessment

The exposure of interest in this study encompassed seven urinary OH-PAHs, which were measured at the NHANES Mobile Examination Center (MEC). These included 1-Hydroxynaphthalene (1-OHNa), 2-Hydroxynaphthalene (2-OHNa), 3-Hydroxyfluorene (3-OHFlu), 2-Hydroxyfluorene (2-OHFlu), 1-Hydroxyphenanthrene (1-OHPh), 1-Hydroxypyrene (1-OHP), and 2–3-Hydroxyphenanthrene (2–3-OHPh). Urinary PAH metabolites were quantified using high-performance liquid chromatography-tandem mass spectrometry (HPLC–MS/MS) employed in the 2013–2016 cycles^26^. The lower limits of detection (LLOD) for these metabolites were as follows: 1-OHNa at 60 ng/L, 2-OHNa at 90 ng/L, 3-OHFlu at 8 ng/L, 2-OHFLU at 8 ng/L, 1-OHPh at 9 ng/L, 2–3-OHPh at 10 ng/L, and 1-OHP at 70 ng/L. The total OH-PAH concentration was calculated by summing the concentrations of all seven individual OH-PAH compounds. All individual OH-PAH compounds have detection rates exceeding 95%, except for 1-Hydroxypyrene, which has a detection rate of 70%.

### Covariates

A structured questionnaire administered during a home interview collected sociodemographic information for NHANES participants, including age (in years), sex (male/female), race (Mexican American, Non-Hispanic Black, Non-Hispanic White, Other), and education level (college graduate or above, high school graduate or GED, less than high school, some college or AA) (Mallah et al., 2022b). Body mass index (BMI) was calculated using the CDC cut-off values as the ratio of weight in kilograms to height in meters squared. For this study, adults were categorized based on BMI cut-off values: "under and normal weight" with a BMI of less than 25, "overweight" with a BMI of 25 to less than 30, and "obese" with a BMI of 30 or greater. The poverty-to-income ratio (PIR) served as a measure of household income, calculated by dividing the annual household income by the poverty threshold for the respective family size in the participant's state of residency for a given year, in accordance with federal guidelines^27^. Log-transformed urinary creatinine (UCR) concentration (mg/dL) was used as covariate in the models to adjust for the variation of the urine sample.

### Outcomes

Ischemic heart disease was identified based on affirmative responses to any of the three heart diseases or symptom-related questions (coronary heart disease, angina, and heart attack). These questions were evaluated through personal interviews using a standardized health status questionnaire. Participants were asked, "Has a doctor or other health professional ever told you that you have coronary artery disease/angina/heart attack?". The RDW was incorporated into the complete blood count (CBC), which was analyzed using a Beckman Coulter MAXM Instrument (Beckman Coulter Inc. Brea, California). This device calculates CBC parameters, such as RDW, based on the Beckman Coulter method, which involves counting, sizing, and automatic dilution and mixing for sample processing.

### Statistical analysis

To satisfy the normality assumption of the models, continuous variables such as OH-PAH, RDW, and creatinine were log-transformed prior to modeling. Sample characteristics were reported as mean and standard deviation (SD) following log-transformation, while categorical variables were expressed as counts (n) and percentages (%). P-values were derived using Student's t-tests or chi-square tests. Logistic regression models were developed to assess the associations between PAH metabolites and IHD, incorporating sampling weights to generate unbiased estimates and more accurate standard errors. Multiple linear regression models were constructed to evaluate the relationship between PAH metabolites and RDW. To facilitate the interpretation of OH-PAH effects on RDW, coefficients were converted to the percentage change in RDW when OH-PAH concentration increased two-fold. Both crude and adjusted models were constructed to evaluate whether the effects were attenuated by covariates. The crude model was developed without incorporating any covariates (Table S2–S3), while the adjusted models included the covariates to account for potential confounding factors. The Benjamini-Hochberg (BH) adjustment method was employed to address the potential increase in Type I error due to multiple testing, effectively controlling the false discovery rate and enhancing the reliability and robustness of the findings^28^.

The Weighted Quantile Sum (WQS) regression model was employed to create a weighted index for estimating the mixed effects associated with all predictors on an outcome. In this study, we assumed β1 to be positive and fitted a Gaussian distributed linear function. We randomly split the data into a training dataset (40%) and a validation dataset (60%) to estimate the weight of the WQS index in the training set. The “gWQS” package facilitated our Weighted Quantile Sum (WQS) regression.

To evaluate the mediating effects of RDW on the association between PAH metabolites and IHD, we conducted a mediation analysis adjusted for covariates. The mediation analysis was carried out using a sequence of models to evaluate each respective effect. Total effects were assessed by coefficient c from model 1:

$$logit\left({P}_{IHD=1}\right)=c\times PAH+ \beta \times covariates+ {e}_{1},$$

where ${P}_{IHD=1}$ is probability of having IHD, covariates were age, sex, race, education, PIR, obesity, UCR; $\beta$ is the coefficients term for covariates; ${e}_{1}$ is the error term. Effect of predictor on mediator was assessed by coefficient a from the model 2:

$$RDW=a\times PAH+ \gamma \times covariates+ {e}_{2},$$

where $\gamma$ is the coefficient of the covariates; ${e}_{2}$ is the error term. The effect of the mediator on outcome controlling for predictor can be assessed from the coefficients b of the model 3:

$$logit\left({P}_{IHD=1}\right)={c}{\prime}\times PAH+b\times RDW+ \delta \times covariates+ {e}_{3},$$

where $\delta$ is the coefficient of the covariates. Given that the product of the coefficients often yields small values, we multiplied all the effects by 1000 to facilitate clearer interpretation. As a result, the mediation effect presented in our findings is scaled accordingly. The indirect effect, representing mediation, is derived from the product of the outcomes from models 2 and 3, calculated as $1000\times a\times b$. The relative effects were calculated as the percentage of indirect or direct effects divided by the total effects. For mediation analysis, we utilized the “mediation” R package.

Bayesian Kernel Machine Regression (BKMR) analysis was employed to examine the potential non-linear and interactive effects of PAH metabolites on IHD and RDW outcomes. This method allowed us to capture the complex and joint effects of multiple OH-PAHs while accounting for potential confounding factors^29^. BKMR analysis was performed using R statistical software with the “bkmr” package^30^. The analysis included the same covariates as in the logistic and multiple linear regression models, to ensure consistency and comparability of results. Model convergence was assessed using trace plots and the Gelman-Rubin diagnostic^31^. Posterior inclusion probabilities (PIPs) were computed to quantify the strength of the association between each PAH metabolite and the outcomes, providing insight into the most influential PAH compounds^30^. All p-values were adjusted using the BH method, and a p-value less than 0.05 was considered statistically significant. All analyses were conducted using the statistical software R 4.2.1^32^.

## Results

### Characteristics of participants

In the analytic sample of 3,003 participants from NHANES (Table 1), 180 (6%) had ischemic heart disease (IHD), and 2,823 (94%) were without IHD. Participants with IHD had a significantly lower proportion of college graduates (23.3% vs. 26.3%, p = 0.0208) and a higher proportion of males (62.8% vs. 48%, p < 0.001) compared to those without IHD. Race/ethnicity distribution significantly differed between IHD and non-IHD patients (p < 0.001), with a higher percentage of non-Hispanic Whites in the IHD group (56.1% vs. 38.6%). IHD patients were older (64.9 ± 12.5 vs. 47.24 ± 16.87, p < 0.001) and had lower mean PIR (2.1 ± 1.45 vs. 2.54 ± 1.64, p < 0.001). IHD patients exhibited higher PAH metabolite concentrations (p < 0.05) and mean RDW (2.65 ± 0.08 vs. 2.61 ± 0.08, p < 0.001). No significant differences were found in obesity distribution, 2-OHNa, 1-OHP, and UCR between groups (p > 0.05).

**Table 1:** General sample characteristics of study participants by ischemic heart disease (IHD) Status (n = 3003).

Categorical variable	All (n = 3003)	IHD (n = 180)	Non-IHD (n = 2823)	
	N (%)			p value
Education				
College graduate or above	785(26.1)	42(23.3)	743(26.3)	0.0208
High school graduate or GED	666(22.2)	52(28.9)	614(21.7)	
Less than high school	632(21)	45(25)	587(20.8)	
Some college or AA	920(30.6)	41(22.8)	879(31.1)	
Sex				
Female	1534(51.1)	67(37.2)	1467(52)	< 0.001
Male	1469(48.9)	113(62.8)	1356(48)	
Obesity				
Normal	863(28.7)	41(22.8)	822(29.1)	0.1785
Obesity	1169(38.9)	74(41.1)	1095(38.8)	
Overweight	971(32.3)	65(36.1)	906(32.1)	
Race/Ethnicity				
Mexican American	452(15.1)	14(7.8)	438(15.5)	< 0.001
Non-Hispanic Black	587(19.5)	33(18.3)	554(19.6)	
Non-Hispanic White	1191(39.7)	101(56.1)	1090(38.6)	
Other	773(25.7)	32(17.8)	741(26.2)	
Continuous variable	Mean (SD)			
Age	48.3(17.16)	64.9(12.5)	47.24(16.87)	< 0.001
PIR	2.51(1.63)	2.1(1.45)	2.54(1.64)	< 0.001
Total OH-PAH	9.1(1.23)	9.35(1.19)	9.08(1.23)	0.0041
1-OHNa	7.5(1.57)	8(1.56)	7.47(1.56)	< 0.001
2-OHNa	8.53(1.17)	8.68(1.1)	8.52(1.17)	0.068
3-OHFlu	4.51(1.41)	4.89(1.46)	4.49(1.4)	< 0.001
2-OHFlu	5.34(1.24)	5.72(1.28)	5.31(1.23)	< 0.001
1-OHPh	4.62(0.95)	4.81(0.88)	4.61(0.96)	0.0028
1-OHP	4.84(0.89)	4.93(0.94)	4.84(0.88)	0.2000
2–3-OHPh	4.84(0.99)	5.04(0.99)	4.83(0.99)	0.0046
RDW	2.61(0.08)	2.65(0.08)	2.61(0.08)	< 0.001
UCR	4.51(0.74)	4.56(0.6)	4.51(0.75)	0.2868

### Association of urinary PAH with IHD

In our study, we investigated the association between urinary PAH metabolite concentrations and IHD using logistic regression models adjusted for NHANES sampling weights (Table 2). Crude and adjusted odds ratios (ORs) were estimated for each PAH metabolite, with ORs representing the change in risk of IHD associated with a twofold increase in the original (non-log-transformed) PAH concentrations. In the crude models, significant associations were observed between IHD and several PAH metabolites, including 1-OHNa, 3-OHFlu, 2-OHFlu, 1-OHPh, 1-OHP, and 2–3-OHPh. After adjusting for covariates, significant associations persisted for 1-OHNa, 2-OHNa, 3-OHFlu, 2-OHFlu, 1-OHPh, 1-OHP, and 2–3-OHPh. Detailed statistics, including odds ratios and confidence intervals, are provided in the Table 2.These findings suggest that increased urinary PAH metabolite concentrations, with a twofold increase in the original OH-PAH levels, are significantly associated with a higher risk of IHD, both in unadjusted and adjusted models.

**Table 2:** Adjusted odds ratios for the association between urinary OH-PAH metabolite concentrations and ischemic heart disease (IHD).

Models adjusted for covariates							
	Coef	LCI	UCI	OR	OR LCI	OR UCI	p value
1-OHNa	0.144	0.032	0.256	1.1	1.02	1.19	0.0224
2-OHNa	0.416	0.178	0.653	1.33	1.13	1.57	0.0045
3-OHFlu	0.4	0.232	0.568	1.32	1.17	1.48	< 0.001
2-OHFlu	0.502	0.31	0.694	1.42	1.24	1.62	< 0.001
1-OHPh	0.446	0.158	0.734	1.36	1.12	1.66	0.0088
1-OHP	0.59	0.282	0.898	1.51	1.22	1.86	0.0037
2–3-OHPh	0.478	0.21	0.747	1.39	1.16	1.68	0.0045

“Coef.”, estimated regression coefficient; “LCI” and “UCI”, lower and upper 95% confidence intervals; “OR”, odds ratio with corresponding lower and upper 95% CIs; “p value”, significance adjusted by “BH” method. Adjusted models account for confounders (age, sex, race, education, PIR, obesity, and UCR).

### Association of urinary OH-PAH with RDW

Using linear regression models, we also examined the relationship between log-transformed PAH metabolite concentrations and RDW, a measure of red blood cell size variability (Table 3). The crude and adjusted models estimated the percentage change in RDW associated with a twofold increase in the original (non-log-transformed) OH-PAH concentrations. In the crude models, RDW showed significant positive associations with various PAH metabolites, including 1-OHNa, 2-OHNa, 3-OHFlu, 2-OHFlu, 1-OHPh, 1-OHP, and 2–3-OHPh. After adjusting for covariates, significant associations persisted for 2-OHNa (0.46% change, 95% CI: 0.20–0.72%, p = 0.0027), 3-OHFlu (0.32% change, 95% CI: 0.14–0.51%, p = 0.0028), 2-OHFlu (0.36% change, 95% CI: 0.14–0.58%, p = 0.0052), 1-OHPh (0.30% change, 95% CI: 0.04–0.57%, p = 0.0369), 1-OHP (0.56% change, 95% CI: 0.29–0.83%, p < 0.001), and 2–3-OHPh (0.51% change, 95% CI: 0.24–0.78%, p = 0.0017). The association for 1-OHNa became non-significant after adjusting for covariates (0.14% change, 95% CI: 0.01–0.26%, p = 0.0528). Our findings indicate that increased urinary PAH metabolite concentrations are associated with higher RDW levels, suggesting greater red blood cell size variability.

**Table 3:** Percentage change in RDW associated with a 2-fold increase in urinary PAH metabolite concentrations.

Models adjusted for covariates							
	Coef	LCI	UCI	RDW change (%)	LCI of RDW change (%)	LCI of RDW change (%)	p value
1-OHNa	0.002	0	0.004	0.14	0.01	0.26	0.0528
2-OHNa	0.007	0.003	0.01	0.46	0.20	0.72	0.0027
3-OHFlu	0.005	0.002	0.007	0.32	0.14	0.51	0.0028
2-OHFlu	0.005	0.002	0.008	0.36	0.14	0.58	0.0052
1-OHPh	0.004	0.001	0.008	0.30	0.04	0.57	0.0369
1-OHP	0.008	0.004	0.012	0.56	0.29	0.83	< 0.001
2–3-OHPh	0.007	0.004	0.011	0.51	0.24	0.78	0.0017

“Coef.”, estimated regression coefficient; “LCI” and “UCI”, lower and upper 95% confidence intervals; “RDW Change (%)”, percentage change in RDW with corresponding lower and upper 95% CIs; “p value”, significance adjusted by “BH” method. Adjusted models account for confounders (age, sex, race, education, PIR, obesity, and UCR).

### WQS Regression Analysis for PAH Exposure and Its Relationship with IHD and RDW

The Weighted Quantile Sum (WQS) regression was employed to evaluate the combined effects of the OH-PAH mixture on IHD and RDW (Table 4). For IHD, the crude WQS analysis showed a significant positive association with the OH-PAH mixture (OR: 1.16, 95% CI: 1.06–1.26, p < 0.001). After adjusting for covariates, the association remained significant (OR: 1.23, 95% CI: 1.09–1.37, p < 0.001). For RDW, the crude WQS analysis also revealed a significant positive association with the OH-PAH mixture, and a 1-unit increase in the WQS index resulted in a 0.31% increase in RDW (95% CI: 0.17–0.46, p < 0.001). In the adjusted model, the association persisted but had a smaller effect size than the crude model, with a 1-unit increase in the WQS index corresponding to a 0.23% increase in RDW (95% CI: 0.01–0.44, p = 0.0408).

**Table 4:** Combined effects of OH-PAH mixture on IHD and RDW: weighted quantile sum regression analysis.

WQS analysis							
	Estimate	SE	t value	OR	OR LCL	OR UCL	p value
OH-PAH on IHD	0.2032	0.0578	3.51	1.23	1.09	1.37	< 0.001
OH-PAH on RDW	0.0023	0.0011	2.05	0.23	0.01	0.44	0.0408

“Estimate”, WQS index; “SE”, standard error; “t value”, t-statistic; “OR”, odds ratio (IHD); “OR LCL”/”OR UCL”, 95% CI for odds ratio; “PCT”, RDW change (%); “PCT LCL”/”PCT UCL”, 95% CI for RDW change; “p value”, BH-adjusted significance. Models were adjusted for confounders (age, sex, race, education, PIR, obesity, UCR).

**Figure 2:**
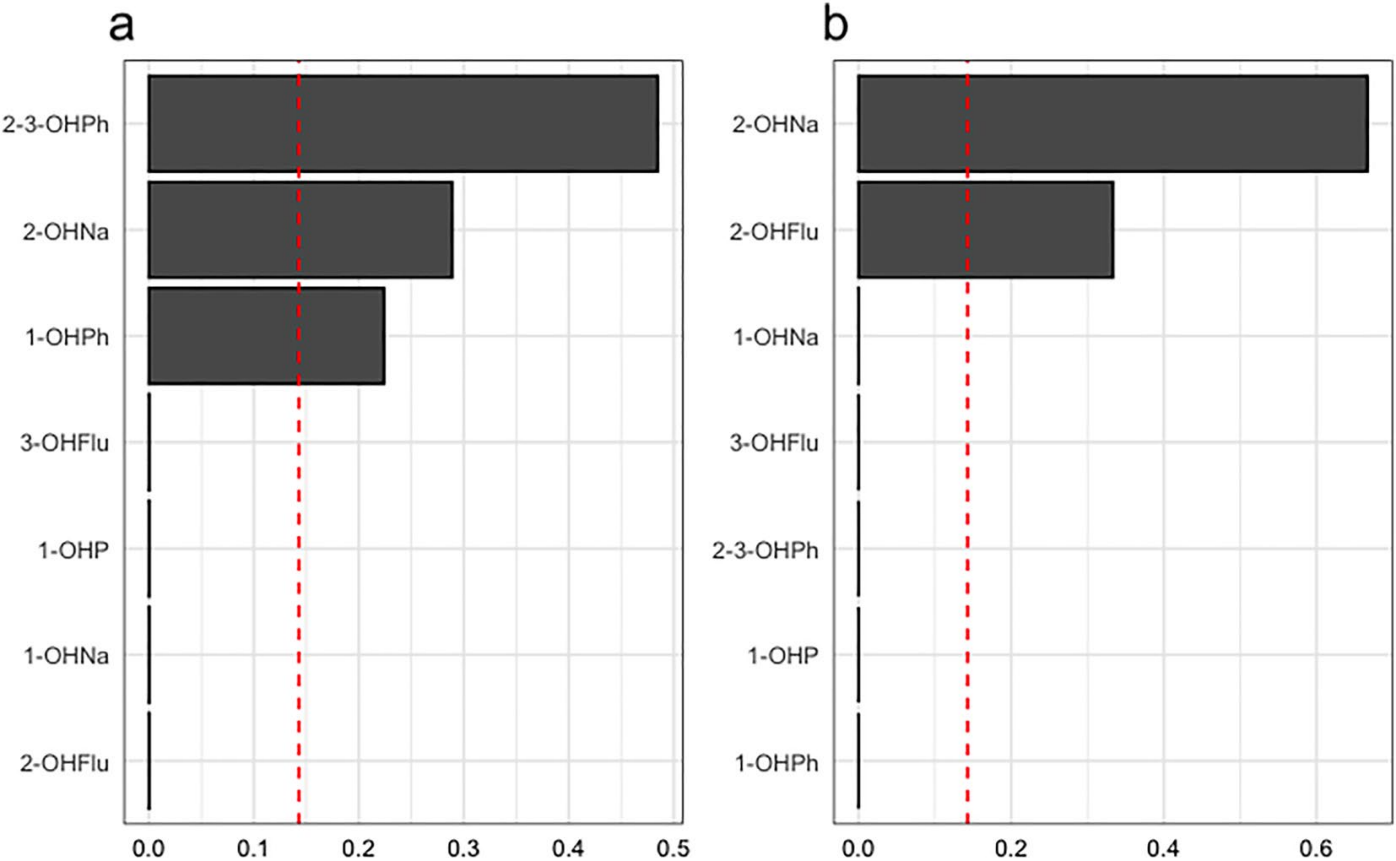
WQS model regression index weights for the RDW and IHD. Models were adjusted for age, sex, race, education, family PIR, obesity, BMI, and urinary creatinine. The red reference lines indicate the cut-off to distinguish elements with significant weights greater than zero. By default, the cut-off is set to the inverse of the number of elements in the mixture.

Figure 2 shows the mean weights of the various PAH metabolites of WQS regressions for RDW (Fig. 2a) and IHD (Fig. 2b). For RDW, 2–3-OHPh (48.47%), 2-OHNa (28.89%), and 1-OHPh (22.40%) exhibited higher mean weights, indicating their greater influence on the relationship with RDW compared to the other PAH metabolites. For IHD, 2-OHNa (66.66%) and 2-OHFlu (33.34) exhibited higher mean weights, indicating their greater influence on the relationship with IHD compared to the other PAH metabolites. These WQS analysis results indicate that the combined exposure to the PAH mixture is significantly associated with an increased risk of IHD and elevated RDW levels, both in unadjusted and adjusted models.

### Mediation effects of PAHs on IHD with RDW as mediators

The results of the mediation analysis investigating the association between PAHs and IHD are presented in Table 5 (adjusted for covariates). Both total PAHs and individual PAHs demonstrate a positive indirect effect, contributing to 2–4.6% of the total effects. Of all the models, total PAHs display the most substantial indirect effect, accounting for 4.6% of total effects (adjusted p-value = 0.016). Regarding individual PAHs, all show significant mediation effects (indirect effects). The highest proportion of the indirect effect contributing to the total effect is found in 1-OHNa (3.9%, p = 0.0284), followed by 2-OHNa (3.5%, p-value = 0.0274) and 2–3-OHPh (3.5%, p-value = 0.0274). In the unadjusted results detailed in Table S5, the indirect effect for Total PAHs contributes to 15.6% of the overall effect. However, for individual PAHs accounting for 7.5–26.9% of their respective total effects. The attenuated indirect effect observed in the adjusted model, as shown in Table 5, is likely due to the inclusion of confounding variables that might have influenced the association between PAH exposure and IHD. In conclusion, the mediation analysis indicates that RDW partially mediates the relationship between PAH exposure and heart disease, suggesting that red blood cell variability could be a contributing factor in the association between PAH exposure and cardiovascular health.

**Table 5:** Mediation analysis of RDW in the relationship between PAH exposure and ischemic heart disease.

	Type	Effect	SE	LCI	UCI	PCT	p value
Total OH-PAH	Indirect	0.101	0.08	0.031	0.469	4.6	0.016
	Direct	2.084	0.414	1.077	2.648	95.3	< 0.001
	Total	2.186	0.449	1.12	2.81	100	< 0.001
1-OHNa	Indirect	0.123	0.081	0.022	0.361	3.9	0.0284
	Direct	3.003	0.44	1.148	3.305	96.1	0.0111
	Total	3.126	0.421	1.396	3.42	100	0.0111
2-OHNa	Indirect	0.047	0.052	0.013	0.321	3.5	0.0274
	Direct	1.277	0.539	0.519	2.604	96.5	< 0.001
	Total	1.324	0.575	0.541	2.815	100	< 0.001
3-OHFlu	Indirect	0.118	0.076	0.027	0.349	2.2	0.0274
	Direct	5.314	0.502	4.243	6.183	97.8	< 0.001
	Total	5.432	0.519	4.325	6.339	100	< 0.001
2-OHFlu	Indirect	0.069	0.052	0.016	0.255	2	0.0274
	Direct	3.353	0.66	2.163	4.666	98	< 0.001
	Total	3.422	0.685	2.186	4.805	100	< 0.001
1-OHPh	Indirect	0.143	0.139	-0.01	0.646	2.6	0.162
	Direct	5.281	1.081	0.901	5.719	97.4	0.0198
	Total	5.424	1.055	1.589	5.92	100	0.0198
1-OHP	Indirect	0.137	0.112	0.03	0.555	2.8	0.0334
	Direct	4.787	0.731	2.651	5.528	97.2	0.0137
	Total	4.924	0.748	2.863	5.782	100	0.0048
2–3-OHPh	Indirect	0.178	0.142	0.043	0.705	3.5	0.0274
	Direct	4.877	0.854	2.498	5.442	96.5	0.0111
	Total	5.056	0.846	2.794	5.645	100	0.0048

“Effect type”, the type of effect (indirect, direct, or total); “Effect value”, the estimated effect value for each type in mediation analysis; “SE”, standard error; LCI”/”UCI”, 95% confidence interval limits; Proportion of Total Effect (%)”, percentage of an effect accounting for the total effect; “Adj. p value”, BH-adjusted significance.

### Association between PAH and IHD, as well as PAH and RDW using the BKMR Model

**Figure 3:**
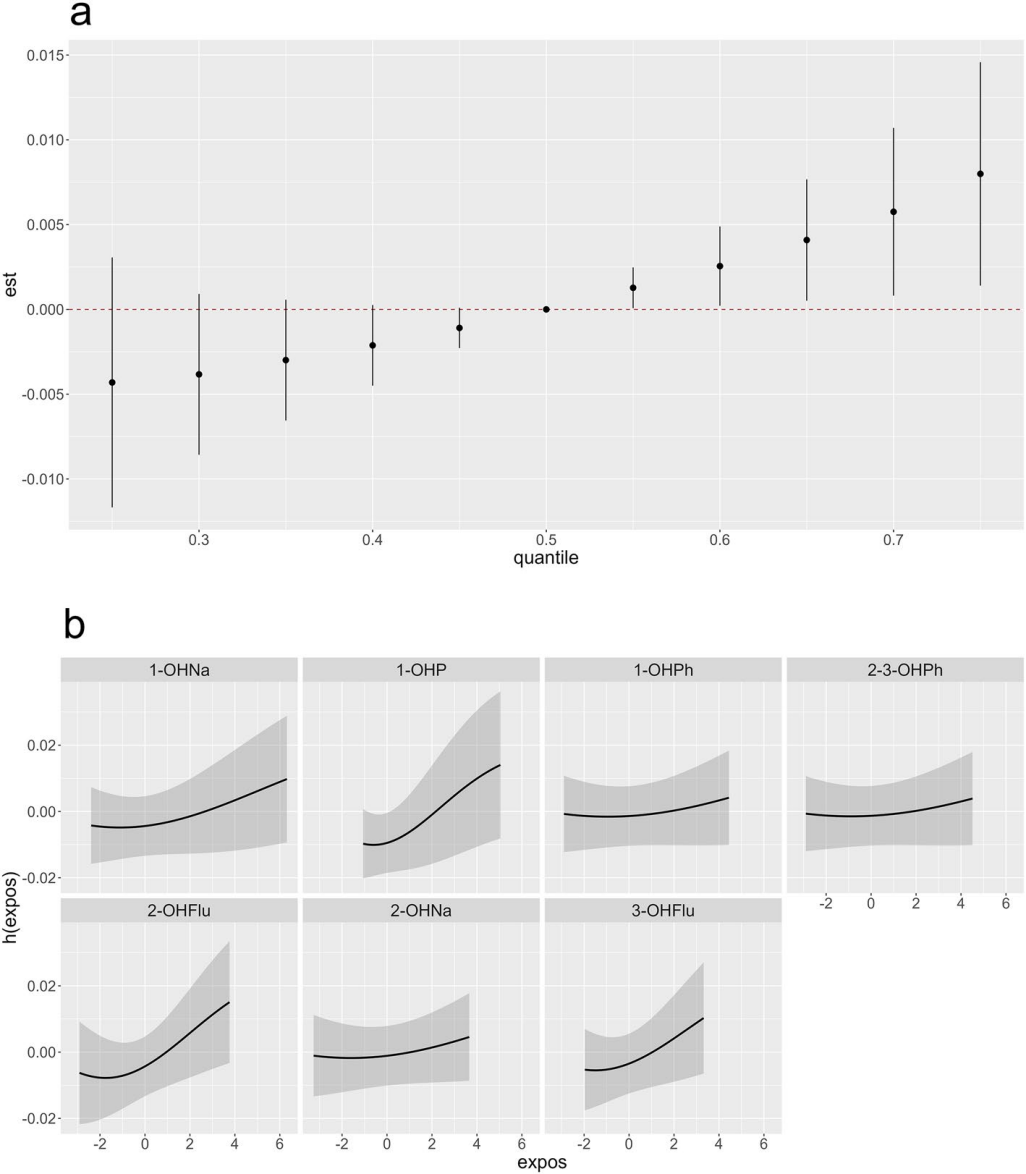
Associations between IHD and scaled, log-transformed OH-PAHs as determined by BKMR analysis. All BKMR models accounted for age, BMI, sex, race, PIR, obesity, and urinary creatinine. (**a**) The combined effects of the mixture on IHD, comparing various percentiles of the mixture to its median values. Y-axis represents the summary risk estimates of developing IHD when all OH-PAHs are fixed at specific quantiles (ranging from 0.25 to 0.75) compared to when OH-PAHs are at the 50th percentile. (**b**) Individual exposure–response functions (h(expos)) and 95% confidence intervals for each OH-PAH (z scores after log-transformation), while maintaining other compounds at their median values.

**Figure 4:**
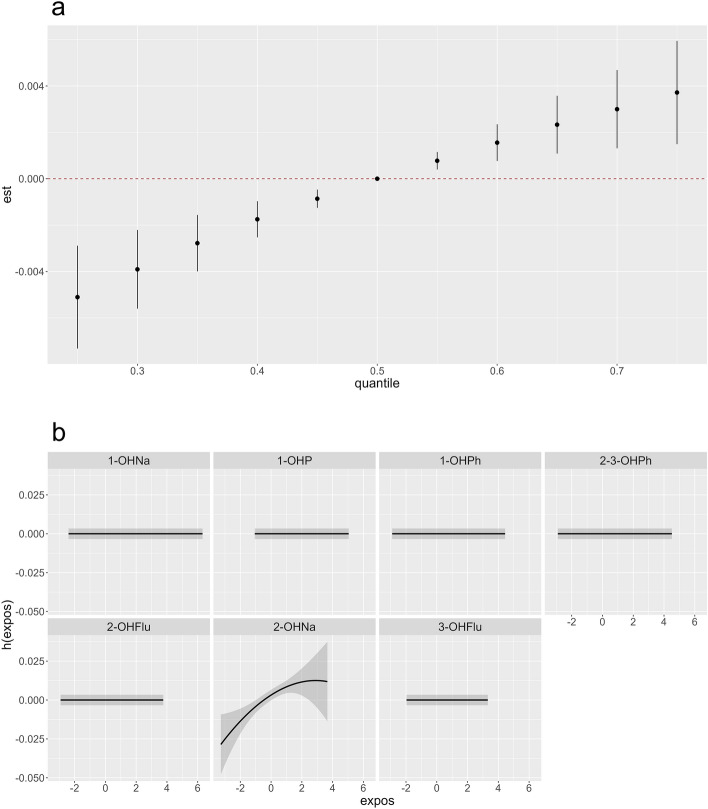
Associations between RDW and scaled, log-transformed OH-PAHs as determined by BKMR analysis. All BKMR models accounted for age, BMI, sex, race, PIR, obesity, and urinary creatinine. (**a**) The combined effects of the mixture on RDW, comparing various percentiles of the mixture to its median values. The Y-axis represents the summary risk estimates for the change in log-transformed RDW when all OH-PAHs are fixed at specific quantiles (ranging from 0.25 to 0.75) compared to when OH-PAHs (z scores after log-transformation) are at the 50th percentile. (**b**) Individual exposure–response functions (h(expos)) and 95% confidence intervals for each OH-PAH, while maintaining other compounds at their median values.

BKMR analysis showed a significant positive overall trend between the concentration of the mixture and the IHD when all the OH-PAH were higher than 55th percentiles (Fig. 3a). After examining the univariate exposure–response functions of the 7 OH-PAHs, we found that all PAH compounds have increasing trends on IHD when other OH-PAHs were held at their median level (Fig. 3b). The BKMR analysis reviewed a significant positive overall trend between the OH-PAH mixture and RDW at OH-PAH between 25 and 75th percentiles (Fig. 4a). Only 2-OHNa showed increasing trends in RDW when all other OH-PAH held at their median level (Fig. 4b).

## Discussion

In this study, we aimed to investigate the relationship between PAH exposure, RDW, and IHD, hypothesizing that RDW may mediate the effect of OH-PAHs on IHD. Our analyses revealed that increased urinary PAH metabolite concentrations were significantly associated with a higher risk of IHD and elevated RDW levels, both in unadjusted and adjusted models. Moreover, our mediation analysis indicated that RDW partially mediates the relationship between PAH exposure and IHD, supporting our hypothesis.

Our results are consistent with previous studies that have reported associations between PAH exposure and increased risk of cardiovascular diseases^16–18^. These studies have suggested that PAH exposure may lead to various cardiovascular effects, including alterations in heart rate variability (HRV), increased risk of IHD, and fatal cardiovascular outcomes. The association between PAH exposure and IHD persisted even after adjusting for potential confounders in our study, emphasizing the potential role of PAHs in the development of IHD. Additionally, the results of our WQS regression analysis further support the significant combined effects of the PAHs mixture on IHD and RDW. This method allowed us to investigate the combined impact of multiple PAHs, as opposed to the individual PAHs in isolation. The WQS results indicate that specific PAH metabolites may have a more significant influence on the relationship between PAH exposure and IHD or RDW. These findings suggest that combined exposure to PAHs may be more relevant to cardiovascular health than individual exposure to specific PAHs.

To the best of our knowledge, our study is among the first to investigate the association between PAH exposure and RDW using a comprehensive dataset representing a national population. While only a few studies have examined this association in animal models or case studies with small sample sizes, our findings offer valuable insights. Zhi et al. (2022) observed no significant impact of PAH-treated oysters on rat RDW-CV^33^, while Booker & White (2005) reported a dose–response relationship between Benzo(a)pyrene (BaP) and RDW in mice exposed to varying concentrations of BaP^34^. In a study of 53 petrochemical plant workers, Wang et al. (2015) found significant non-parametric Spearman correlations between urinary 1-OHP concentration and red cell indices MCH and RDW^35^. Intriguingly, Adu et al. (2018) reported that chronic exposure to petrochemicals might lead to reduced hematopoietic output, including lower RDW and reticulocyte counts in workers^36^. However, the reduced RDW should be considered in the context of the significantly decreased reticulocyte count.

In our study, we discovered that increased urinary PAH metabolite concentrations were associated with higher RDW levels. Although the precise mechanisms linking RDW to cardiovascular diseases remain uncertain, factors such as inflammation, oxidative stress, and impaired iron metabolism have been proposed as potential contributors (Danese et al., 2015). RDW has been associated with inflammation and higher levels of inflammatory cytokines, such as IL-6, IL-8, and TNF-alpha^37^. Increased RDW levels have also been linked to oxidative stress, which often accompanies chronic inflammation and can decrease the lifespan of red blood cells^38^. Our findings support the hypothesis that PAH exposure may contribute to these underlying processes, resulting in elevated RDW levels and, consequently, an increased risk of cardiovascular diseases.

While our mediation analysis confirms RDW's role in the link between PAH exposure and IHD, it further highlights the significance of RDW as a potential biomarker for cardiovascular diseases. The mechanisms driving the association between PAH exposure, RDW, and cardiovascular health is not fully understood yet, warranting further exploration. The quantified indirect effect, specified as 2–3.9% for individual PAHs and 4.6% for cumulative PAH exposure, while seemingly modest, holds significant practical implications. Such instances, where an indirect effect accounts for a small portion of the total effect yet holds significant implications, are not uncommon in mediation analysis literature^39–41^. Given the widespread prevalence of PAH exposure, these percentages can have marked clinical implications at the population level. Small or modest indirect effects can accumulate over time or in conjunction with other mediators, leading to significant outcomes. In the context of PAH exposure and IHD, such sustained influences, even if minor, might have a substantial cumulative impact on cardiovascular health. Additionally, even modest indirect effects, when consistently linked to the outcome variable, play a pivotal role in deciphering underlying mechanisms, offering invaluable understanding of the intricate pathways and interactions shaping observed results. The mediation by RDW provides a glimpse into potential biological pathways, suggesting that PAH exposure might influence erythrocyte variability, thereby shaping cardiovascular outcomes. This hypothesis, while promising, demands rigorous examination in follow-up studies. It's also vital to acknowledge that PAH may influence heart disease through a multitude of other yet unidentified pathways. From a clinical standpoint, this mediation insight furnishes healthcare professionals with an enriched perspective: intensive RDW monitoring could be beneficial for those with heightened PAH exposure, serving as a precursor for cardiovascular risks. Furthermore, interventions tailored to mitigate the PAH's impact on RDW might emerge as strategic preventive measures against IHD.

Our study has some limitations that should be noted. First, the cross-sectional nature of the NHANES data precludes us from making causal inferences between PAH exposure, RDW, and IHD. Longitudinal studies are needed to confirm the temporal relationship between these factors. Second, we relied on single-spot urine samples to measure PAH metabolite concentrations, which may partially capture long-term exposure. Future studies should consider using multiple samples or other biomarkers to better estimate PAH exposure. Lastly, residual confounding may still be present despite adjusting for potential confounders in our analyses. In addition to the limitations mentioned above, our study also presents several strengths. First, we utilized a large, nationally representative sample from NHANES, which enhances the generalizability of our findings to the U.S. population. Second, our study employed rigorous statistical analyses, including WQS regression and mediation analysis, allowing us to assess the combined effects of PAH exposure and its potential mediator RDW. These advanced analytical techniques contribute to a more comprehensive understanding of the relationship between PAH exposure, RDW, and IHD.

## Conclusions

In conclusion, our study provides novel insights into the relationship between PAH exposure, RDW, and IHD. The findings highlight the potential role of RDW as a mediator in the association between PAH exposure and cardiovascular health, emphasizing the importance of addressing environmental pollutants like PAHs to reduce the burden of cardiovascular diseases. Further research should validate these findings and investigate the underlying mechanisms that link PAH exposure, RDW, and IHD.

### Supplementary Information

Supplementary Information.

## Data Availability

The datasets used and/or analyzed during the current study are available from the corresponding author upon reasonable request.

## References

[1] Ravindra K, Sokhi R, Van Grieken R (2008). Atmospheric polycyclic aromatic hydrocarbons: Source attribution, emission factors and regulation. Atmos. Environ..

[2] Hagy, J. D., Kurtz, J. C. & Greene, R. M. *An approach for developing numeric nutrient criteria for a Gulf coast estuary. US Environmental Protection Agency, Office of Research and Development, National Health and Environmental Effects Research Laboratory, Research Triangle Park, NC*. (2008).

[3] Beyer J, Jonsson G, Porte C, Krahn MM, Ariese F (2010). Analytical methods for determining metabolites of polycyclic aromatic hydrocarbon (PAH) pollutants in fish bile: A review. Environ. Toxicol. Pharmacol..

[4] Dong C-D, Chen C-F, Chen C-W (2012). Determination of polycyclic aromatic hydrocarbons in industrial harbor sediments by GC-MS. Int. J. Environ. Res. Public Health.

[5] Veltman K, Huijbregts MA, Rye H, Hertwich EG (2011). Including impacts of particulate emissions on marine ecosystems in life cycle assessment: The case of offshore oil and gas production. Integr. Environ. Assess. Manag..

[6] Kim K-H, Jahan SA, Kabir E, Brown RJC (2013). A review of airborne polycyclic aromatic hydrocarbons (PAHs) and their human health effects. Environ. Int..

[7] Li Z (2021). Global, regional, and national death, and disability-adjusted life-years (DALYs) for cardiovascular disease in 2017 and trends and risk analysis from 1990 to 2017 using the global burden of disease study and implications for prevention. Front. Public Health.

[8] Roth GA (2020). Global burden of cardiovascular diseases and risk factors, 1990–2019: Update from the GBD 2019 study. J. Am. Coll. Cardiol..

[9] Yusuf S (2004). Effect of potentially modifiable risk factors associated with myocardial infarction in 52 countries (the INTERHEART study): Case–control study. The Lancet.

[10] Bozorgi A (2016). Red cell distribution width and severe left ventricular dysfunction in ischemic heart failure. Crit. Pathways Cardiol. J. Evid. Based Med..

[11] Ruckerl R (2006). Air pollution and markers of inflammation and coagulation in patients with coronary heart disease. Am. J. Respir. Crit. Care Med..

[12] Scarborough P, Allender S, Rayner M, Goldacre M (2012). Contribution of climate and air pollution to variation in coronary heart disease mortality rates in England. PLoS ONE.

[13] Ma J (2022). Longitudinal relationships between polycyclic aromatic hydrocarbons exposure and heart rate variability: Exploring the role of transforming growth factor-β in a general Chinese population. J. Hazard. Mater..

[14] Yu J, Fang Q, Liu M, Zhang X (2021). Polycyclic aromatic hydrocarbons associated long non-coding RNAs and heart rate variability in coke oven workers. Environ. Sci. Pollut. Res..

[15] Zhang Y, Du W, Yang B (2019). Long non-coding RNAs as new regulators of cardiac electrophysiology and arrhythmias: Molecular mechanisms, therapeutic implications and challenges. Pharmacol. Therap..

[16] Mallah MA (2022). Association of urinary polycyclic aromatic hydrocarbon metabolites and cardiovascular disease among US population: A cross-sectional study. Environ. Res..

[17] Burstyn I (2005). Polycyclic aromatic hydrocarbons and fatal ischemic heart disease. Epidemiology.

[18] Costello S, Garcia E, Hammond SK, Eisen EA (2013). Ischemic heart disease mortality and PM3.5 in a cohort of autoworkers. Am. J. Ind. Med..

[19] Allen LA (2010). Validation and potential mechanisms of red cell distribution width as a prognostic marker in heart failure. J. Cardiac Fail..

[20] Al-Najjar Y, Goode KM, Zhang J, Cleland JG, Clark AL (2009). Red cell distribution width: An inexpensive and powerful prognostic marker in heart failure. Eur. J. Heart Fail..

[21] Danese E, Lippi G, Montagnana M (2015). Red blood cell distribution width and cardiovascular diseases. J. Thorac. Dis..

[22] Isik T (2012). Relation of red cell distribution width with the presence, severity, and complexity of coronary artery disease. Coron. Artery Dis..

[23] Zalawadiya SK, Veeranna V, Niraj A, Pradhan J, Afonso L (2010). Red cell distribution width and risk of coronary heart disease events. Am. J. Cardiol..

[24] Tonelli M (2008). Relation between red blood cell distribution width and cardiovascular event rate in people with coronary disease. Circulation.

[25] Centers for Disease Control and Prevention (CDC). National Center for Health Statistics (NCHS). National Health and Nutrition Examination Survey Data. Hyattsville, MD: U.S. Department of Health and Human Services, Centers for Disease Control and Prevention. https://www.cdc.gov/nchs/nhanes/about_nhanes.htm (2023).

[26] Centers for Disease Control and Prevention (CDC). National Health and Nutrition Examination Survey.Data Documentation, Codebook, and Frequencies. https://wwwn.cdc.gov/Nchs/Nhanes/2015-2016/PAH_I.htm (2023).

[27] Cowan AE (2018). Dietary supplement use differs by socioeconomic and health-related characteristics among US adults, NHANES 2011–2014. Nutrients.

[28] Benjamini Y, Hochberg Y (1995). Controlling the false discovery rate: A practical and powerful approach to multiple testing. J. R. Stat. Soc. Ser. B (Methodol.).

[29] Bobb JF (2015). Bayesian kernel machine regression for estimating the health effects of multi-pollutant mixtures. Biostatistics.

[30] Bobb JF, Claus Henn B, Valeri L, Coull BA (2018). Statistical software for analyzing the health effects of multiple concurrent exposures via Bayesian kernel machine regression. Environ. Health.

[31] Gelman, A., Carlin, J. B., Stern, H. S., Dunson, D. B. & Vehtari, A. Rubin DB. Bayesian data analysis. *Chapman &* (2004).

[32] R Core Team, R. R: A language and environment for statistical computing. (2013).

[33] Zhi J (2022). Degradation of curcumin-mediated photodynamic technology (PDT) on polycyclic aromatic hydrocarbons in oysters and toxicity evaluation of PDT-treated oysters. Int. J. Food Sci. Technol..

[34] Booker CD, White KL (2005). Benzo(a)pyrene-induced anemia and splenomegaly in NZB/WF1 mice. Food Chem. Toxicol..

[35] Wang L (2015). Cancer risk of petrochemical workers exposed to airborne PAHs in industrial Lanzhou City, China. Environ. Sci. Pollut. Res..

[36] Adu P (2018). Reduced haematopoietic output in automobile mechanics and sprayers with chronic exposure to petrochemicals: A case–control study in Cape Coast, Ghana. J. Environ. Public Health.

[37] Agarwal S (2012). Red cell distribution width, inflammatory markers and cardiorespiratory fitness: Results from the National Health and Nutrition Examination Survey. Indian Heart J..

[38] Joosse H-J (2023). In-vitro and in-silico evidence for oxidative stress as drivers for RDW. Sci. Rep..

[39] Dregan A (2020). Associations between depression, arterial stiffness, and metabolic syndrome among adults in the UK biobank population study: A mediation analysis. JAMA Psychiatry.

[40] Guo Y (2018). Cardiometabolic traits mediated the relationship from urinary polycyclic aromatic hydrocarbons metabolites to heart rate variability reduction: A community-based study. Environ. Pollut..

[41] Ilango SD (2020). The role of cardiovascular disease in the relationship between air pollution and incident dementia: A population-based cohort study. Int. J. Epidemiol..

