# Supplementary material for: Association between polycyclic aromatic hydrocarbons exposure with red cell width distribution and ischemic heart disease: insights from a population-based study
Source: Sci Rep. 2024 Jan 2;14:196. doi: 10.1038/s41598-023-50794-x (PMC10762247; doi:10.1038/s41598-023-50794-x)
Supplement: Supplementary file 1 — Supplementary Information. [file 41598_2023_50794_MOESM1_ESM.docx]

Supplemental Materials

Figure S1. Flowchart of included participants


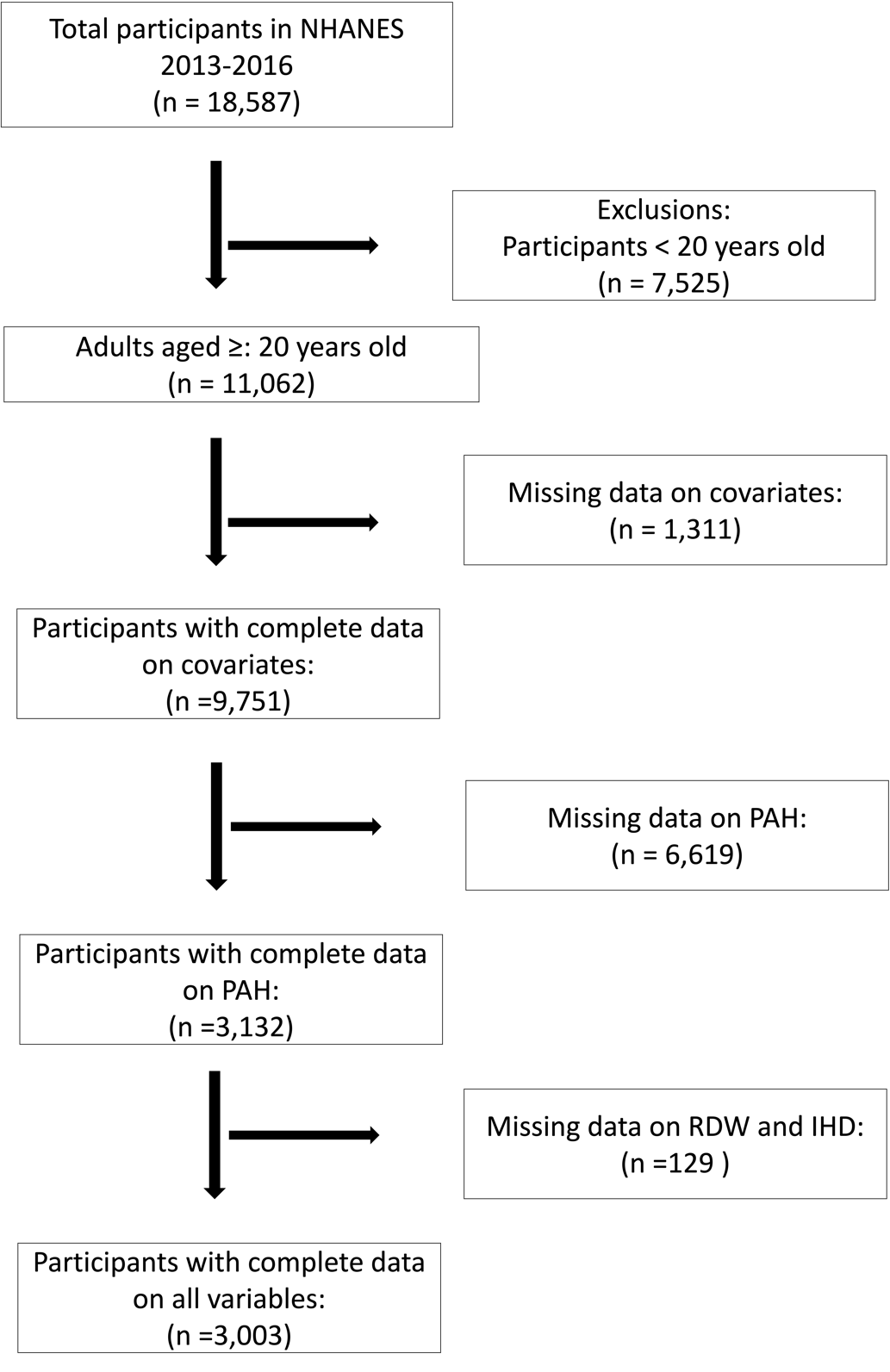


Table S1. Statistical comparison of demographic variables and their distributions between overall and analytic dataset.

| Name of variable | Overall dataset | Analytic dataset | Tests | Test statistics | P values |
| --- | --- | --- | --- | --- | --- |
| Age | 49.3(17.59) | 48.3(17.16) | Kolmogorov-Smirnov test | D = 0.026661 | 0.06963 |
| PIR | 2.46(1.62) | 2.51(1.63) | Kolmogorov-Smirnov test | D = 0.016943 | 0.5202 |
| Education |  |  |  |  |  |
| College graduate or above | 2766(25.0) | 785(26.1) | Pearson's Chi-squared test | X-squared = 3.9687, df = 3 | 0.2133 |
| High school graduate or GED | 2443(22.1) | 666(22.2) |  |  |  |
| Less than high school | 2502(22.6) | 632(21) |  |  |  |
| Some college or AA | 3343(30.2) | 920(30.6) |  |  |  |
| Race |  |  |  |  |  |
| Mexican American | 1700(15.4) | 452(15.1) | Pearson's Chi-squared test | X-squared = 6.25, df = 3 | 0.1001 |
| Non-Hispanic Black | 2308(20.9) | 587(19.5) |  |  |  |
| Non-Hispanic White | 4188(37.9) | 1191(39.7) |  |  |  |
| Other | 2866(25.9) | 773(25.7) |  |  |  |
| Gender |  |  |  |  |  |
| Female | 5769(52.2) | 1534(51.1) | Pearson's Chi-squared test | X-squared = 0.043381, df = 1 | 0.835 |
| Male | 5293(47.8) | 1469(48.9) |  |  |  |

Note: Mean and standard deviation have been calculated for age and PIR. Categorical variables were displayed by showing both the count of participants in each category and the proportion they represented. Comparisons between overall and analytic datasets were made exclusively for adults (aged 20 and older).

Table S2: Crude Odds Ratios for the Association between Urinary OH-PAH Metabolite Concentrations and Ischemic Heart Disease (IHD)

| **Crude models** | | | | | | | |
| --- | --- | --- | --- | --- | --- | --- | --- |
|  | Coef. | LCI | UCI | OR | OR LCI | OR UCI | p value |
| 1-OHNa | 0.185 | 0.107 | 0.262 | 1.14 | 1.08 | 1.2 | <0.001 |
| 2-OHNa | 0.141 | -0.01 | 0.291 | 1.1 | 0.99 | 1.22 | 0.0779 |
| 3-OHFlu | 0.231 | 0.115 | 0.347 | 1.17 | 1.08 | 1.27 | <0.001 |
| 2-OHFlu | 0.293 | 0.167 | 0.42 | 1.23 | 1.12 | 1.34 | <0.001 |
| 1-OHPh | 0.324 | 0.198 | 0.45 | 1.25 | 1.15 | 1.37 | <0.001 |
| 1-OHP | 0.252 | 0.088 | 0.415 | 1.02 | 1.01 | 1.04 | 0.0062 |
| 2-3-OHPh | 0.331 | 0.195 | 0.466 | 1.26 | 1.14 | 1.38 | <0.001 |

Abbreviations: “Coef.”, estimated regression coefficient; “LCI” and “UCI”, lower and upper 95% confidence intervals; “OR”, odds ratio with corresponding lower and upper 95% CIs; “p value”, significance adjusted by “BH” method. Crude models are unadjusted.

Table S3: Crude Percentage Change in RDW Associated with a 2-Fold Increase in Urinary PAH Metabolite Concentrations

| **Crude model** | | | | | | | |
| --- | --- | --- | --- | --- | --- | --- | --- |
|  | Coef. | LCI | UCI | RDW Change (%) | LCI of RDW Change (%) | LCI of RDW Change (%) | p value |
| 1-OHNa | 0.004 | 0.001 | 0.006 | 0.25% | 0.10% | 0.40% | 0.003 |
| 2-OHNa | 0.008 | 0.005 | 0.011 | 0.53% | 0.33% | 0.74% | <0.001 |
| 3-OHFlu | 0.004 | 0.002 | 0.007 | 0.31% | 0.14% | 0.48% | 0.0014 |
| 2-OHFlu | 0.005 | 0.003 | 0.008 | 0.38% | 0.18% | 0.58% | <0.001 |
| 1-OHPh | 0.006 | 0.002 | 0.009 | 0.41% | 0.17% | 0.65% | 0.0022 |
| 1-OHP | 0.007 | 0.003 | 0.011 | 0.07% | 0.03% | 0.11% | 0.0018 |
| 2-3-OHPh | 0.007 | 0.004 | 0.011 | 0.51% | 0.26% | 0.77% | <0.001 |

Abbreviations: “Coef.”, estimated regression coefficient; “LCI” and “UCI”, lower and upper 95% confidence intervals; “RDW Change (%)”, percentage change in RDW with corresponding lower and upper 95% CIs; “p value”, significance adjusted by “BH” method. Crude models are unadjusted.

Table S4. Crude combined Effects of OH-PAH Mixture on IHD and RDW: Weighted Quantile Sum Regression Analysis.

| WQS analysis of OH-PAH on IHD | | | | | | | |
| --- | --- | --- | --- | --- | --- | --- | --- |
|  | Estimate | SE | t value | OR | OR LCL | OR UCL | p value |
| OH-PAH on IHD | 0.1475 | 0.0435 | 3.39 | 1.16 | 1.06 | 1.26 | <0.001 |
| OH-PAH on RDW | 0.0031 | 0.0007 | 4.21 | 0.31 | 0.17 | 0.46 | <0.001 |

Abbreviations: “Estimate”, WQS index; “SE”, standard error; “t value”, t-statistic; “OR”, odds ratio (IHD); “OR LCL”/”OR UCL”, 95% CI for odds ratio; “PCT”, RDW change (%); “PCT LCL”/”PCT UCL”, 95% CI for RDW change; “p value”, BH-adjusted significance. All models are crude model without adjusting for covariates.

Table S5. Mediation Analysis of RDW in the Relationship between PAH Exposure and Ischemic Heart Disease (crude models).

|  | Type | Effect | SE | LCI | UCI | PCT | P value |
| --- | --- | --- | --- | --- | --- | --- | --- |
| Total OH-PAH | Indirect | 0.374 | 0.218 | 0.180 | 1.206 | 15.6 | <0.001 |
|  | Direct | 2.020 | 0.521 | 0.960 | 2.302 | 84.4 | 0.010 |
|  | Total | 2.394 | 0.484 | 1.435 | 2.825 | 100.0 | 0.006 |
| 1-OHNa | Indirect | 0.246 | 0.095 | 0.127 | 0.531 | 8.4 | <0.001 |
|  | Direct | 2.685 | 0.264 | 2.119 | 3.151 | 91.6 | <0.001 |
|  | Total | 2.931 | 0.300 | 2.305 | 3.455 | 100.0 | <0.001 |
| 2-OHNa | Indirect | 0.729 | 0.435 | 0.318 | 2.389 | 26.9 | <0.001 |
|  | Direct | 1.976 | 1.493 | -9.179 | 2.160 | 73.0 | 0.209 |
|  | Total | 2.706 | 1.180 | -6.065 | 2.753 | 100.0 | 0.080 |
| 3-OHFlu | Indirect | 0.419 | 0.152 | 0.221 | 0.842 | 8.2 | <0.001 |
|  | Direct | 4.705 | 0.688 | 2.722 | 5.389 | 91.8 | 0.008 |
|  | Total | 5.124 | 0.635 | 3.419 | 5.833 | 100.0 | 0.006 |
| 2-OHFlu | Indirect | 0.326 | 0.136 | 0.170 | 0.726 | 7.5 | <0.001 |
|  | Direct | 4.009 | 0.426 | 3.127 | 4.649 | 92.5 | 0.004 |
|  | Total | 4.335 | 0.444 | 3.340 | 5.011 | 100.0 | <0.001 |
| 1-OHPh | Indirect | 0.541 | 0.228 | 0.246 | 1.208 | 10.3 | <0.001 |
|  | Direct | 4.700 | 0.772 | 0.878 | 5.235 | 89.7 | 0.006 |
|  | Total | 5.241 | 0.672 | 2.514 | 5.809 | 100.0 | 0.006 |
| 1-OHP | Indirect | 0.769 | 0.377 | 0.381 | 2.085 | 19.3 | <0.001 |
|  | Direct | 3.210 | 3.010 | -16.541 | 4.385 | 80.7 | 0.304 |
|  | Total | 3.979 | 2.752 | -14.876 | 4.964 | 100.0 | 0.217 |
| 2-3-OHPh | Indirect | 0.591 | 0.254 | 0.298 | 1.459 | 11.9 | <0.001 |
|  | Direct | 4.393 | 0.862 | 0.118 | 4.823 | 88.1 | 0.012 |
|  | Total | 4.985 | 0.736 | 2.167 | 5.507 | 100.0 | 0.008 |

Abbreviations: “Effect type”, the type of effect (indirect, direct, or total); “Effect value”, the estimated effect value for each type in mediation analysis; “SE”, standard error; “LCI”/”UCI”, 95% confidence interval limits; “Proportion of Total Effect (%)”, percentage of an effect accounting for the total effect; “Adj. p value”, BH-adjusted significance.
